# Low rather than high mean corpuscular volume is associated with mortality in Japanese patients under hemodialysis

**DOI:** 10.1038/s41598-020-72765-2

**Published:** 2020-09-24

**Authors:** Hirokazu Honda, Miho Kimachi, Noriaki Kurita, Nobuhiko Joki, Masaomi Nangaku

**Affiliations:** 1 Department of Medicine, Division of Nephrology, Showa University School of Medicine, 1-5-8, Hatanodai, Shinagawa-ku, Tokyo, 142-8666 Japan; 2 Department of Healthcare Epidemiology, School of Public Health in the Graduate School of Medicine, Koto University, Kyoto, Japan; 3 Institute for Health Outcomes and Process Evaluation Research (iHope International), Kyoto, Japan; 4 Department of Clinical Epidemiology, Graduate School of Medicine, Fukushima Medical University, Fukushima, Japan; 5 Department of Innovative Research and Education for Clinicians and Trainees (DiRECT), Fukushima Medical University Hospital, Fukushima, Japan; 6 Center for Innovative Research for Communities and Clinical Excellence (CiRC2LE), Fukushima Medical University, Fukushima, Japan; 7 Division of Nephrology, Toho University Ohashi Medical Center, Tokyo, Japan; 8 Division of Nephrology and Endocrinology, The University of Tokyo, Tokyo, Japan

**Keywords:** Nephrology, Haemodialysis

## Abstract

Recent studies have reported that high mean corpuscular volume (MCV) might be associated with mortality in patients with advanced chronic kidney disease (CKD). However, the question of whether a high MCV confers a risk for mortality in Japanese patients remains unclear. We conducted a longitudinal analysis of a cohort of 8571 patients using data derived from the Japan Dialysis Outcomes and Practice Patterns Study (J-DOPPS) phases 1 to 5. Associations of all-cause mortality, vascular events, and hospitalization due to infection with baseline MCV were examined via Cox proportional hazard models. Non-linear relationships between MCV and these outcomes were examined using restricted cubic spline analyses. Associations between time-varying MCV and these outcomes were also examined as sensitivity analyses. Cox proportional hazard models showed a significant association of low MCV (< 90 fL), but not for high MCV (102 < fL), with a higher incidence of all-cause mortality and hospitalization due to infection compared with 94 ≤ MCV < 98 fL (reference). Cubic spline analysis indicated a graphically U-shaped association between baseline MCV and all-cause mortality (p for non-linearity p < 0.001). In conclusion, a low rather than high MCV might be associated with increased risk for all-cause mortality and hospitalization due to infection among Japanese patients on hemodialysis.

## Introduction

Anemia is a common complication of chronic kidney disease (CKD) and an important risk factor for mortality, especially in patients with advanced CKD^1–6^. Efforts to identify associations between anemia and mortality have focused on the impact of low hemoglobin, hemoglobin variability and hypo-responsiveness to erythropoiesis-stimulating agents (ESA)^1–6^. Recent studies have indicated that larger erythrocytes, estimated as an increase in mean corpuscular volume (MCV), are associated with increased risk for all-cause and cardiovascular (CVD) mortality in patients with CKD regardless of whether or not they are on dialysis^7–10^.

MCV is variously associated with the development of microcytic, normocytic and macrocytic types of anemia. Macrocytic anemia is associated with a vitamin B12 or folate deficiency, alcohol consumption, hypothyroidism and liver disease^11–14^, whereas microcytic anemia is associated with thalassemia, iron deficiency, lead poisoning and chronic inflammation^15,16^. Thus, several factors influence the size of erythrocytes. However, the iron status of Japanese and non-Japanese patients differs under hemodialysis due to different management strategies. Ferritin is managed at lower levels in patients on hemodialysis in Japan than in other countries^17^. Differences in iron status might influence the size of erythrocytes and MCV distribution, resulting in different associations between MCV and clinical outcomes in patients on hemodialysis. Indeed, both iron deficiency and iron surplus have been associated with increased mortality in Japanese patients^18–20^.

Accordingly, we postulated that low rather than high MCV is a risk factor for mortality among Japanese patients on hemodialysis. Here, we assessed these associations in a cohort of such patients.

## Results

### Study flow diagram and baseline characteristics

**Figure 1:**
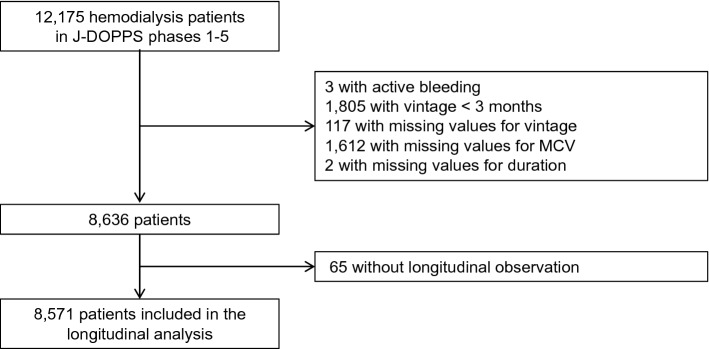
Participant flow diagram and study selection process. *MCV* mean corpuscular volume.

A total of 12,175 hemodialysis patients were included in the Japan Dialysis Outcomes and Practice Patterns Study (J-DOPPS) phases 1 to 5. Figure 1 shows the study flow diagram and study selection process. After exclusion of 3539 patients who met exclusion criteria described in the “Materials and methods” section and 65 patients without longitudinal observation, 8571 patients were included in the longitudinal analysis.

As shown in Table 1, MCV was categorized as low in 16.3% (n = 1401), slightly low in 17.9% (n = 1535), medium (reference) in 24.5% (n = 2100), slightly high in 21.3% (n = 1828), and high in 19.9% (n = 1707). Among all participants, mean age was 62.5 (standard deviation [SD] 12.7) years, 37.2% were female, and median MCV was 96.1 fL. Patients with a high MCV tended to be older, more frequently female, have a longer dialysis vintage, have greater use of ESAs, and have higher levels of transferrin saturation (TSAT) and ferritin. Patients with low MCV tended to be younger, more frequently male, have high white blood cell counts, have no use of ESA, and have lower levels of TSAT and ferritin. Hemoglobin levels among the five categories were closely similar.

**Table 1:** Baseline characteristics of patients.

	Total (n = 8571)	MCV < 90 fL (n = 1401)	90 ≤ MCV < 94 fL (n = 1535)	94 ≤ MCV < 98 fL (n = 2100)	98 ≤ MCV < 102 fL (n = 1828)	102 ≤ MCV fL (n = 1707)	p-value
Age, years	62.5 (12.7)	60.2 (12.6)	61.2 (12.6)	62.1 (12.7)	63.4 (12.5)	65.3 (12.5)	< 0.01
Female, %	37.2	31.8	35.7	37.1	38.5	41.7	< 0.01
BMI	20.9 (3.3)	21.4 (3.5)	21.2 (3.2)	21.0 (3.2)	20.8 (3.2)	20.2 (3.1)	< 0.01
Dialysis vintage, year	5.1 (1.8–10.6)	4.6 (1.2–10.7)	4.5 (1.4–9.5)	5.0 (1.7–10.3)	5.2 (2.0–10.9)	5.7 (2.3–11.4)	< 0.01
**Primary renal disease, %**							< 0.01
Chronic glomerulonephritis	45.8	39.8	39.2	45.5	49.0	53.2	
Nephrosclerosis	5.2	4.2	4.5	5.7	5.3	5.9	
Diabetic nephropathy	29.9	35.9	36.5	30.4	26.6	22.1	
Secondary glomerulonephritis	1.5	1.7	1.2	1.3	1.6	1.6	
Interstitial nephritis	2.3	2.5	2.4	2.1	2.7	2.1	
Others	15.4	15.9	16.3	15.1	14.9	15.2	
**Comorbidities, %**							
Diabetes mellitus	33.0	38.8	39.7	33.4	29.4	25.7	< 0.01
Cardiovascular diseases	29.6	31.1	28.3	29.3	27.9	31.8	0.054
Cerebrovascular diseases	13.8	14.6	12.8	13.2	13.5	14.9	0.34
Peripheral vascular diseases	14.9	16.9	15.1	14.0	13.8	15.2	0.11
COPD	2.6	3.3	2.2	2.2	2.7	2.6	0.33
Liver cirrhosis	2.6	2.4	2.5	2.6	1.9	3.8	0.25
Cancer	9.0	8.1	8.7	9.2	9.2	9.4	0.74
**ESAs, %**							< 0.01
rHuEPO-α (or β)	66.9	56.5	63.7	67.5	69.7	74.0	
Darbepoetin-α	18.1	21.8	20.5	17.6	16.6	15.5	
Epoetin-β pegol	1.9	2.1	2.8	1.3	1.7	2.0	
Not used	13.1	19.6	13.0	13.6	12.1	8.5	
**ERI**							
rHuEPO-α (or β), IU/week/kg/g/dL	16.3 (7.2–37.3)	17.4 (8.5–49.5)	14.7 (7.0–35.8)	15.3 (6.7–34.5)	16.7 (7.3–35.5)	17.5 (7.6–38.3)	< 0.01
Darbepoetin-α, μg/week/kg/g/dL	0.042 (0.026–0.071)	0.054 (0.032–0.092)	0.042 (0.025–0.063)	0.039 (0.025–0.063)	0.037 (0.023–0.062)	0.046 (0.025–0.075)	< 0.01
Epoetin-β pegol, μg/week/kg/g/dL	0.046 (0.030–0.067)	0.053 (0.039–0.067)	0.045 (0.032–0.069)	0.045 (0.030–0.068)	0.043 (0.029–0.054)	0.044 (0.029–0.072)	0.64
Intravenous iron, %	27.4	28.8	26.1	24.1	28.9	29.9	< 0.01
**Laboratory variables**							
MCV, fL	96.1 (6.9)	85.4 (4.0)	91.9 (1.1)	95.7 (1.1)	99.5 (1.1)	105.4 (3.1)	< 0.01
Hemoglobin, g/L	10.2 (1.3)	10.1 (1.5)	10.3 (1.3)	10.3 (1.3)	10.3 (1.3)	10.1 (1.3)	< 0.01
WBC, 10^3^/mm^3^	6.0 (1.9)	6.4 (2.1)	6.1 (2.0)	5.9 (1.9)	5.8 (1.8)	5.6 (1.9)	< 0.01
TSAT, %	24.4 (11.5)	17.9 (11.6)	22.7 (11.1)	25.4 (10.6)	26.9 (10.9)	28.8 (10.3)	< 0.01
Ferritin, ng/mL	112.7 (44.2–260)	58 (20.4–154.5)	90 (35.6–200)	110 (47–238)	140 (58–310.1)	166 (84.5–376)	< 0.01
CRP, mg/dL	0.12 (0.06–0.38)	0.17 (0.08–0.47)	0.11 (0.05–0.30)	0.15 (0.06–0.40)	0.10 (0.05–0.26)	0.13 (0.06–0.46)	< 0.01

Results of continuous variables are shown as mean (SD) or median (interquartile range).

*BMI* body mass index, *COPD* chronic obstructive pulmonary disease, *ESA* erythropoiesis-stimulating agents, *ERI* erythropoiesis resistance index, *rHuEPO-α* (*or β*) recombinant human erythropoietin-alpha (or beta), *MCV* mean corpuscular volume, *WBC* white blood cell, *TSAT* transferrin saturation, *CRP* C-reactive protein.

ERI was calculated by dividing the weekly ESA doses by body weight (kg) per hemoglobin value (g per dL).

Further, 1731 patients with missing values for MCV, vintage and study duration tended to be slightly older. Compared to those without missing values at baseline, these patients were more frequently female; and had a longer hemodialysis vintage; a lower frequency of chronic glomerulonephritis and higher frequency of diabetic nephropathy as primary renal disease; a higher frequency of diabetes mellitus, liver cirrhosis, and cancer as comorbidities; a lower frequency of cardiovascular disease and COPD as comorbidities; and greater use of darbepoetin-α or epoetin-β pegol (see Supplement Table 1). In addition to MCV values, these patients also frequently had missing data for other laboratory variables.

### Association between MCV and mortality

**Figure 2:**
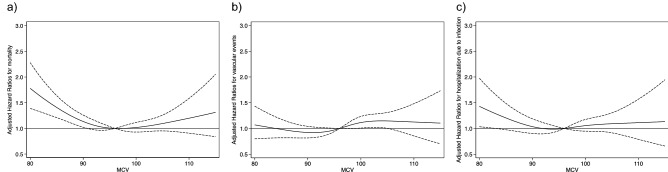
Nonlinear relationship between MCV values and outcomes. *MCV* mean corpuscular volume. Figures show nonlinear relationship between MCV and outcomes (**a**) all-cause mortality, (**b**) vascular events, and (**c**) hospitalization due to infection. Models were adjusted for all potential risk factors at baseline, including age, gender, body mass index, dialysis vintage, primary renal diseases, comorbidities (diabetes mellitus, cardiovascular diseases, cerebrovascular diseases, peripheral atherosclerotic vascular diseases, chronic obstructive pulmonary disease, liver cirrhosis, and cancer), laboratory measurements (levels of hemoglobin, white blood cells, transferrin saturation, ferritin, and C-reactive protein), and the use of erythropoiesis-stimulating agents and intravenous iron infusion.

The median follow-up period was 2.1 years (inter-quartile range [IQR] 1.5–2.7 years). A total of 911 (10.6%) patients (low 180 [12.9%]; slightly low 131 [8.5%]; medium 203 [9.7%]; slightly high 189 [10.3%]; high 208 [12.2%]) died during the observation period (Supplement Table 2). No significant differences among MCV categories were noted in the causes of all-cause death (p = 0.08). The low MCV group had a significantly higher incidence of all-cause mortality than the medium MCV group as reference in the Cox proportional hazard model. In contrast, no significant difference in incidence was noted among the other groups (see Table 2). Continuous association between MCV and all-cause mortality estimated via restricted cubic spline analysis was U-shaped (p for non-linearity < 0.001) (see Fig. 2), and the risk for all-cause mortality began to increase from MCV 90 fL to 80  fL.

**Table 2:** Association between MCV and all-cause mortality.

	Model 1 h	p-value	Model 2 h	p-value	Model 3 h	p-value
Low (MCV < 90 fL)	1.41 (1.14–1.75)	0.001	1.41 (1.14–1.74)	0.001	1.39 (1.09–1.78)	0.009
Slightly low (90 ≤ MCV < 94 fL)	0.90 (0.72–1.12)	0.34	0.89 (0.72–1.11)	0.32	0.89 (0.72–1.10)	0.28
Medium (94 ≤ MCV < 98 fL)	Reference		Reference		Reference	
Slightly high (98 ≤ MCV < 102 fL)	1.06 (0.87–1.28)	0.58	1.05 (0.87–1.28)	0.59	0.96 (0.79–1.16)	0.65
High (102 ≤ MCV fL)	1.27 (1.04–1.55)	0.02	1.27 (1.04–1.54)	0.02	1.01 (0.83–1.24)	0.90

Results are shown as HR with 95% confidence intervals.

Adjusted for Model 1 (minimally adjusted model): levels of ferritin, transferrin saturation (TSAT), Model 2: levels of ferritin, TSAT and C-reactive protein (CRP), Model 3 (fully adjusted model): all potential risk factors at baseline, including age, gender, body mass index, dialysis vintage, primary renal diseases, comorbidities (diabetes mellitus, cardiovascular diseases, cerebrovascular diseases, peripheral atherosclerotic vascular diseases, chronic obstructive pulmonary disease, liver cirrhosis, and cancer), laboratory values (hemoglobin, white blood cells, TSAT, ferritin and CRP), administration of erythropoiesis-stimulating agents and intravenous iron infusion.

*HR* hazard ratio, *MCV* mean corpuscular volume.

A sensitivity analysis using a time-varying Cox regression model was conducted using 4-month interval data from 51,785 measurements of 8539 patients. Findings from the analysis between time-varying MCV values and mortality were closely consistent with the original results (see Supplement Table 3). Nonlinear relationship estimated via the restricted cubic spline model were also revealed to be U-shaped (p for non-linearity < 0.001) (see Supplement Fig. 1).

### Association between MCV and vascular events

A total of 758 (8.8%) patients (low 115 [8.2%]; slightly low 129 [8.4%]; medium 194 [9.2%]; slightly high 159 [8.7%]; high 161 [9.4%]) experienced vascular events during the observation period. No significant difference in the incidence of vascular events was noted among the low or high MCV groups compared to reference group (see Table 3). Further, no significant nonlinear association estimated via restricted cubic spline analysis was noted between MCV and vascular events (p for non-linearity 0.13) (see Fig. 2).

**Table 3:** Association between MCV and vascular events.

	Model 1 h	p-value	Model 2 h	p-value	Model 3 h	p-value
Low (MCV < 90 fL)	0.93 (0.76–1.13)	0.44	0.93 (0.76–1.13)	0.44	0.89 (0.73–1.10)	0.28
Slightly low (90 ≤ MCV < 94 fL)	0.92 (0.74–1.13)	0.41	0.92 (0.74–1.13)	0.41	0.89 (0.72–1.10)	0.28
Medium (94 ≤ MCV < 98 fL)	Reference		Reference		Reference	
Slightly high (98 ≤ MCV < 102 fL)	0.93 (0.78–1.12)	0.46	0.93 (0.78–1.12)	0.46	0.97 (0.81–1.17)	0.77
High (102 ≤ MCV fL)	1.05 (0.83–1.32)	0.70	1.05 (0.83–1.32)	0.69	1.09 (0.88–1.34)	0.44

Results are shown as HR with 95% confidence intervals.

Adjusted for Model 1 (minimally adjusted model): levels of ferritin, transferrin saturation (TSAT), Model 2: levels of ferritin, TSAT and C-reactive protein (CRP), Model 3 (fully adjusted model): all potential risk factors at baseline, including age, gender, body mass index, dialysis vintage, primary renal diseases, comorbidities (diabetes mellitus, cardiovascular diseases, cerebrovascular diseases, peripheral atherosclerotic vascular diseases, chronic obstructive pulmonary disease, liver cirrhosis, and cancer), laboratory values (hemoglobin, white blood cells, TSAT, ferritin and CRP), administration of erythropoiesis-stimulating agents and intravenous iron infusion.

*HR* hazard ratio, *MCV* mean corpuscular volume.

A sensitivity analysis using a time-varying Cox regression model was conducted using 4-month interval data from 49,680 measurements of 8534 patients. Similar to the original results, no significant difference in the incidence of vascular events was noted among time-varying low MCV groups compared to the reference group. In contrast, time-varying high MCV groups revealed a significantly higher incidence of vascular events than the reference groups (see Supplement Table 4). A nonlinear relationship estimated via the restricted cubic spline model showed a lower incidence and higher incidence of vascular events at 90 fL and 100 fL of MCV, respectively (p for non-linearity 0.023) (see Supplement Fig. 1).

### Association between MCV and hospitalization due to infection

A total of 559 (6.5%) patients (low 103 [7.4%]; slightly low 88 [5.7%]; medium 121 [5.8%]; slightly high 119 [6.5%]; high 128 [7.5%]) were hospitalized due to infection during the observation period. The low MCV group had a significantly higher incidence of hospitalization due to infection than the medium MCV group as reference in the Cox proportional hazards model. In contrast, no significant difference in incidence was noted among the other groups (see Table 4). Additionally, no significant nonlinear association estimated via restricted cubic spline analysis was noted between MCV and hospitalization due to infection (p for non-linearity 0.094) (see Fig. 2).

**Table 4:** Association between MCV and hospitalization due to infection.

	Model 1 h	p-value	Model 2 h	p-value	Model 3 h	p-value
Low (MCV < 90 fL)	1.38 (1.03–1.86)	0.033	1.38 (1.03–1.86)	0.033	1.36 (1.02–1.81)	0.037
Slightly low (90 ≤ MCV < 94 fL)	1.02 (0.80–1.30)	0.87	1.02 (0.80–1.30)	0.89	1.01 (0.80–1.28)	0.92
Medium (94 ≤ MCV < 98 fL)	Reference		Reference		Reference	
Slightly high (98 ≤ MCV < 102 fL)	1.11 (0.87–1.42)	0.41	1.11 (0.87–1.42)	0.41	1.04 (0.83–1.32)	0.72
High (102 ≤ MCV fL)	1.30 (1.03–1.64)	0.028	1.30 (1.03–1.64)	0.028	1.12 (0.89–1.42)	0.33

Results are shown as HR with 95% confidence intervals.

Adjusted for Model 1 (minimally adjusted model): levels of ferritin, transferrin saturation (TSAT), Model 2: levels of ferritin, TSAT and C-reactive protein (CRP), Model 3 (fully adjusted model): all potential risk factors at baseline, including age, gender, body mass index, dialysis vintage, primary renal diseases, comorbidities (diabetes mellitus, cardiovascular diseases, cerebrovascular diseases, peripheral atherosclerotic vascular diseases, chronic obstructive pulmonary disease, liver cirrhosis, and cancer), laboratory values (hemoglobin, white blood cells, TSAT, ferritin and CRP), administration of erythropoiesis-stimulating agents and intravenous iron infusion.

*HR* hazard ratio, *MCV* mean corpuscular volume.

A sensitivity analysis using a time-varying Cox regression model was conducted using 4-month interval data from 50,480 measurements of 8534 patients. Similar to the original results, the time-varying low MCV group had a significantly higher incidence of hospitalization due to infection than the reference group. In addition, time-varying high MCV groups revealed significantly higher incidence than the reference groups (see Supplement Table 5). In contrast, the restricted cubic spline model did not support evidence of a non-linear relationship between MCV and hospitalization due to infection (p for non-linearity 0.064) (see Supplement Fig. 1).

## Discussion

In this study, we identified an association of low, rather than high, MCV with all-cause mortality and a higher prevalence of hospitalization due to infection among Japanese patients on hemodialysis by baseline and time-varying Cox regression models. A high MCV was not associated with all-cause mortality by categorized MCV analysis, while continuous association between MCV and all-cause mortality by restricted cubic spline analysis was U-shaped. These findings differ from those of previous studies in non-Japanese patients, in which high MCV was a risk factor for all-cause and CVD-related mortality^9^.

A low MCV is closely associated with an iron deficiency and chronic inflammation^15^. We found that the ferritin value was lowest in the group with a low MCV, whereas C-reactive protein (CRP) values did not differ among MCV groups. Moreover, the low MCV indicated a significant iron shortage in the regression model (Supplement Table 6). The effect of low iron status causing low MCV is closely associated with mortality and an increased prevalence of hospitalization among patients with CKD^18,20,21^. Iron deficiency causes wide distribution in the size of red blood cells [red blood cell distribution width (RDW)]^22^, which might lead to increased mortality in populations on dialysis^23^. Iron deficiency is also associated with diminished mitochondrial function^24,25^, and endothelial^8^ and skeletal muscle^26^ dysfunction, which in turn results in increased rates of heart failure^24^, cardiovascular disease^25^, and sarcopenia due to skeletal muscle myopathy^26^. Moreover, several clinical studies in patients with stroke and CVD have demonstrated similar associations of microcytic anemia with such clinical outcomes as unfavorable functional outcomes; hospitalization and inpatient mortality^27,28^; and restenosis after percutaneous coronary intervention^29^. The absence of an association between MCV and CVD outcomes in our present study is likely explained by the lower incidence of CVD events among Japanese patients on hemodialysis than in other countries. Thus, we speculate that iron status might be a key influence on MCV, and a key determinant of the impact of MCV on mortality and hospitalization among Japanese patients on hemodialysis.

Nevertheless, the observed associations between a low MCV and all-cause mortality independent of ferritin and TSAT in the present study suggest that factors other than biomarkers of iron metabolism may contribute to the association of low MCV with these clinical outcomes. However, it remains unclear why a low MCV per se or concomitant situation other than iron deficiency would cause an increase in clinical events. Possible reasons may be associated with iron administration and utilization of stored iron. The median ferritin value in our cohort (112 ng/mL) was lower than those reported by Dratch et al. (median value 283 ng/dL)^9^ and Tennankore et al. (mean value 729 ng/mL)^8^. In fact, both transferrin saturation (TSAT) and ferritin levels in patients with a low MCV in the present study were clearly lower than those described by Dratch et al., in which TSAT and ferritin levels in groups with MCV < 90 fL were more than 20% and 200 ng/mL, respectively^9^. These differences in iron status may themselves account for differences in the size of erythrocytes and distribution of MCV, given that the median MCV described by Dratch et al. was 93 fL, and thus lower than in our study. Anemia in Japanese patients under hemodialysis is managed with lower levels of ferritin than in other countries and iron administration is restricted to prevent excess administration of iron^17,30^. Hepcidin level decreases in low-ferritin conditions, with the result that iron absorption and utilization of stored iron are increased^31^. In this setting, MCV may be increased by iron administration and/or ESA therapy with concomitant low levels of TSAT and ferritin, given that the 4 groups with MCV > 90 fL in the present study included patients with low levels of TSAT and ferritin (Table 1). Accordingly, the observed associations between MCV and clinical outcomes in the present study were unlikely to be influenced by differences in biomarkers of iron metabolism.

In the present study, a low MCV was associated with iron deficiency. Thus, it is possible that iron supplementation could improve MCV value, resulting in turn in an improvement in clinical outcome. However, we are unable to mention the impact of changes in MCV by iron supplementation on clinical outcome because of the present study design. Further studies regarding the effectiveness of iron administration are needed to determine whether iron administration improves MCV and subsequent clinical outcomes in patients on hemodialysis with low MCV.

Since macrocytosis is associated with poor health status and malnutrition^9,10^ and is frequently accompanied by severe concomitant comorbidities^9^, a large MCV might serve as a surrogate marker for mortality and CVD events. In our present study, a high MCV was associated with CVD-related mortality in a time-varying Cox regression model, but was not predictive of CVD outcomes in the baseline Cox regression model. Thus, a large MCV might be a risk for the development of CVD events over the short term in Japanese patients on hemodialysis. However, the associations of high MCV with all-cause mortality and hospitalization due to infection were not similar to those with CVD events. While a non-linear, U-shaped relationship between MCV and all-cause mortality was evident on cubic spline analysis, categorical analysis revealed no association between high MCV versus medium MCV with increased mortality in either the baseline or time-varying model. With regard to the time-varying model, categorical analysis revealed an association between high MCV versus medium MCV with increased hospitalization for infection, whereas a non-linear relationship between MCV and hospitalization for infection was not evident on cubic spline analysis. This discrepancy might be ascribable to differences in statistical modeling, such as in the choice of thresholds for MCV categories and in the number and place of knots to determine splines for continuous MCV. The high MCV group patients were older and had a lower body mass index (BMI) than those with a low MCV. Although the prevalence of CVD was similar in patients with a high and low MCV, those of cancer and liver cirrhosis were higher in patients with a high MCV. These findings are similar to those of previous studies^8–10^. Overall, a high MCV might potentially be a risk factor for clinical outcome in Japanese patients on hemodialysis.

Moreover, the differences in the association of a large MCV with clinical outcomes between our present study and these previous studies might also be due to treatment with ESA^8,9^. Therapy with ESA might influence the size of MCV and increase reticulocyte counts^8,32,33^ as well as the occurrence of clinical events in patients with CKD^34–36^. High- rather than low-dose short-acting ESA therapy might be a risk factor for mortality among Japanese patients on hemodialysis^18^, albeit that the highest dose in Japan is lower than that in other countries^17^. Moreover, one study found that higher doses of long- rather than short-acting ESA and higher doses of long-acting ESA might be a risk factor for mortality among Japanese patients on hemodialysis^34^. Thus, ESA might affect the association between increased MCV and clinical outcomes of Japanese patients. However, we found here that the ESA response to hemoglobin and the rates of short- and long-acting ESA administration did not differ among the five groups categorized by MCV at baseline. Against this background, associations between ESA dose and MCV size should be validated in an appropriate prospective cohort study.

The present study has some limitations. First, several patients were excluded from analysis because of missing biomarkers. Second, anemia was managed according to the Japanese guidelines for patients with CKD^30^, but the actual method for managing anemia, such as with ESA therapy and ferrotherapy, was decided by the primary physicians. Further study with a larger sample size to confirm the shape of the continuous relationship between MCV and mortality is warranted. Third, we could not precisely assess the association between MCV and each cause of death because of the small sample size. Nevertheless, no apparent significant differences among MCV categories were noted in the causes of all-cause death, including cardiovascular death (Supplement Table 2). Further, the associations of MCV and cardiovascular death or death due to infection showed a similar tendency to the associations of cardiovascular event or hospitalization due to infection (Supplement Table 7–10). Finally, unmeasured covariates did not permit us to examine potential confounding by any of dose of iron supplementation; reported associations of lead, vitamin B12, and folate with MCV size^11,12,37^; or difference in cardiovascular disease and mortality predictability between MCV and RDW among CKD patients^22,38^.

We conclude that low MCV might be associated with increased all-cause mortality and infection-related hospitalization among Japanese patients on hemodialysis.

## Materials and methods

### Design, setting and study participants

This cohort study was based on data from J-DOPPS phases 1 to 5 (phase 1: 1999–2001, phase 2: 2002–2004, phase 3: 2005–2008, phase 4: 2009–2011, phase 5: 2012–2015). Details of DOPPS, including its design, methodology and sampling strategy, have been described elsewhere^39,40^.

Participants were eligible for the present study if they were aged more than 18 years and had been on maintenance hemodialysis for at least 3 months. Patients were excluded if they experienced active bleeding, such as hospitalization due to gastrointestinal bleeding, or blood transfusion in the week before study initiation. Patients were also excluded if they had missing values for MCV.

All participants in J-DOPPS provided informed consent. Our present study was approved by Tokyo Women’s Medical University (approval number: 2338) and complied with the Declaration of Helsinki.

### Outcome measurement

The primary outcome was all-cause mortality during the observational period. Secondary outcomes were vascular events and hospitalization due to infection. Vascular events included hospitalization due to angina, acute myocardial infarction, cardiac arrest, and stroke; performance of cardiac catheterization, coronary angioplasty, and coronary bypass graft (CABG); and death due to acute myocardial infarction and atherosclerotic heart disease. Hospitalization due to infection included pneumonia, bronchitis, septicemia, endocarditis, urinary tract infection, meningitis, cellulitis, osteomyelitis, viral infection, fungal infection, infected access, and procedures for abscess drainage and antibiotic therapy.

### Statistical analyses

The main exposure was MCV level at baseline, which was categorized into five groups at even intervals: MCV < 90 (low), 90 ≤ MCV < 94 (slightly low), 94 ≤ MCV < 98 (reference category, medium), 98 ≤ MCV < 102 (slightly high), and MCV ≥ 102 (high). The reference group included the mean MCV level. Descriptive statistics for participant baseline characteristics were calculated based on MCV categories. Continuous data were summarized as means (SD) for normally distributed variables and medians (IQR) for skewed variables. Dichotomous and categorical data were described as numbers (proportion). Hazard ratios (HRs) were calculated with 95% confidence intervals (95%CIs) to assess the association between MCV and primary or secondary outcomes using the Cox proportional hazard model. We adjusted for levels of ferritin and TSAT in model 1 (minimally adjusted model); levels of ferritin, TSAT and CRP in model 2; and all potential risk factors at baseline, including age; gender; BMI; dialysis vintage; primary renal disease (chronic glomerulonephritis, renal sclerosis, diabetic nephropathy, secondary glomerulonephritis, interstitial nephritis, and others); comorbidities (diabetes mellitus, cardiovascular diseases, cerebrovascular diseases, peripheral atherosclerotic vascular diseases, chronic obstructive pulmonary disease, liver cirrhosis, and cancer); laboratory measurements (levels of hemoglobin, white blood cells (WBC), TSAT, ferritin, and CRP); use of ESAs, categorized into epoetin-α or epoetin-β, darbepoetin-α, epoetin β pegol, and non-use; and administration of intravenous iron in model 3 (fully adjusted model). We used robust standard variance estimation to account for facility clustering.

As sensitivity analyses, we also performed time-varying Cox regression analyses to examine the associations of time-updated MCV and the abovementioned outcomes. Clinical information including laboratory data and medication was collected at 4-month intervals in J-DOPPS (up to nine measurements). We adjusted for time-varying covariates every 4 months, including levels of ferritin and TSAT in model 1 (minimally adjusted model); levels of ferritin, TSAT and CRP in model 2, and baseline characteristics, including patient demographics (age, gender, BMI, dialysis vintage, primary renal diseases) and comorbidities; and for time-varying covariates every 4 months, including laboratory measurements (levels of hemoglobin, WBC, TSAT, ferritin, and CRP) and administration of ESAs and intravenous iron administration in model 3, using robust standard variance estimation to account for facility clustering. With regard to outcome data, we excluded observations made during the first four months.

Additionally, both for baseline Cox hazard models and for time-varying Cox hazard models, we described the restricted cubic spline curve to assess the non-linear relationship between MCV and primary or secondary outcomes. We placed four knots at the 5th, 35th, 65th, and 95th percentiles of MCV, and set MCV = 96 fL as reference^41^.

We performed multiple imputation using chained equations for imputation of missing covariates to create 20 imputed datasets. We then derived the HRs and 95% CIs by combining the results from the multiple imputed datasets based on Rubin’s rule^42^.

All statistical analyses were performed using STATA 16.0 (version 16.0; StataCorp, College Station, TX, USA), with 2-sided significance set at 0.05.

## Supplementary information

Supplementary Figure S1.

Supplementary Table 1.

Supplementary Table 2.

Supplementary Table 3–5.

Supplementary Table 6.

Supplementary Table 7–10.

## Data Availability

The datasets used and/or analysed during the current study are not open-access.
