# Supplementary material for: Low rather than high mean corpuscular volume is associated with mortality in Japanese patients under hemodialysis
Source: Sci Rep. 2020 Sep 24;10:15663. doi: 10.1038/s41598-020-72765-2 (PMC7515877; doi:10.1038/s41598-020-72765-2)
Supplement: Supplementary file 1 — Supplementary Figure S1. [file 41598_2020_72765_MOESM1_ESM.pptx]

## Slide 1
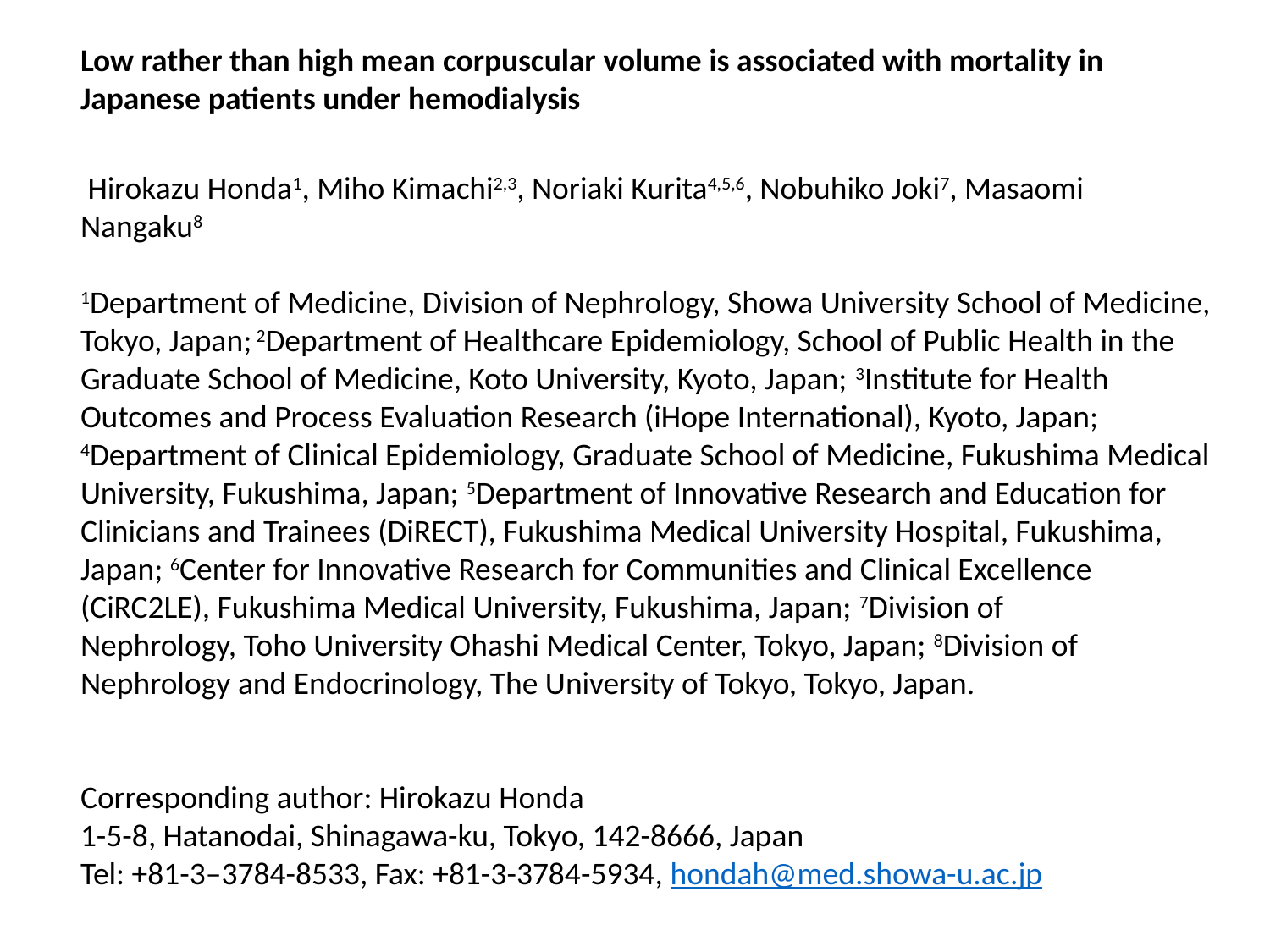

Low rather than high mean corpuscular volume is associated with mortality in Japanese patients under hemodialysis
 Hirokazu Honda1, Miho Kimachi2,3, Noriaki Kurita4,5,6, Nobuhiko Joki7, Masaomi Nangaku8
1Department of Medicine, Division of Nephrology, Showa University School of Medicine, Tokyo, Japan; 2Department of Healthcare Epidemiology, School of Public Health in the Graduate School of Medicine, Koto University, Kyoto, Japan; 3Institute for Health Outcomes and Process Evaluation Research (iHope International), Kyoto, Japan; 4Department of Clinical Epidemiology, Graduate School of Medicine, Fukushima Medical University, Fukushima, Japan; 5Department of Innovative Research and Education for Clinicians and Trainees (DiRECT), Fukushima Medical University Hospital, Fukushima, Japan; 6Center for Innovative Research for Communities and Clinical Excellence (CiRC2LE), Fukushima Medical University, Fukushima, Japan; 7Division of Nephrology, Toho University Ohashi Medical Center, Tokyo, Japan; 8Division of Nephrology and Endocrinology, The University of Tokyo, Tokyo, Japan.
Corresponding author: Hirokazu Honda
1-5-8, Hatanodai, Shinagawa-ku, Tokyo, 142-8666, Japan
Tel: +81-3–3784-8533, Fax: +81-3-3784-5934, hondah@med.showa-u.ac.jp

## Slide 2
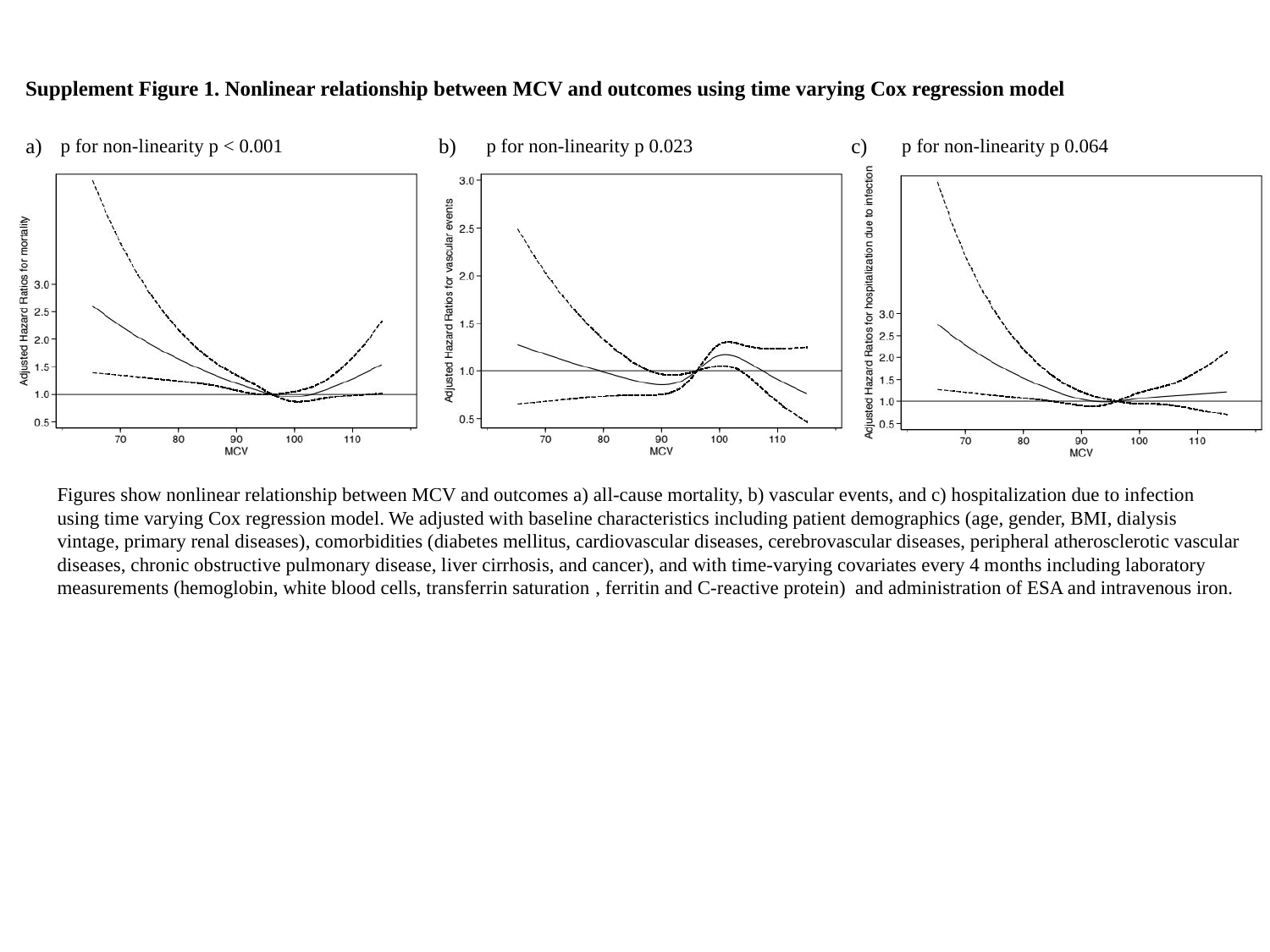

Supplement Figure 1. Nonlinear relationship between MCV and outcomes using time varying Cox regression model
a)
b)
c)
p for non-linearity p < 0.001
p for non-linearity p 0.023
p for non-linearity p 0.064
Figures show nonlinear relationship between MCV and outcomes a) all-cause mortality, b) vascular events, and c) hospitalization due to infection using time varying Cox regression model. We adjusted with baseline characteristics including patient demographics (age, gender, BMI, dialysis vintage, primary renal diseases), comorbidities (diabetes mellitus, cardiovascular diseases, cerebrovascular diseases, peripheral atherosclerotic vascular diseases, chronic obstructive pulmonary disease, liver cirrhosis, and cancer), and with time-varying covariates every 4 months including laboratory measurements (hemoglobin, white blood cells, transferrin saturation , ferritin and C-reactive protein) and administration of ESA and intravenous iron.
