# Supplementary material for: Low rather than high mean corpuscular volume is associated with mortality in Japanese patients under hemodialysis
Source: Sci Rep. 2020 Sep 24;10:15663. doi: 10.1038/s41598-020-72765-2 (PMC7515877; doi:10.1038/s41598-020-72765-2)
Supplement: Supplementary file 2 — Supplementary Table 1. [file 41598_2020_72765_MOESM2_ESM.docx]

**Low rather than high mean corpuscular volume is associated with mortality in Japanese patients under hemodialysis**

Hirokazu Honda^1^, Miho Kimachi^2,3^, Noriaki Kurita^4,5,6^, Nobuhiko Joki^7^, Masaomi Nangaku^8^

^1^Department of Medicine, Division of Nephrology, Showa University School of Medicine, Tokyo, Japan; ^2^Department of Healthcare Epidemiology, School of Public Health in the Graduate School of Medicine, Koto University, Kyoto, Japan; ^3^Institute for Health Outcomes and Process Evaluation Research (iHope International), Kyoto, Japan; ^4^Department of Clinical Epidemiology, Graduate School of Medicine, Fukushima Medical University, Fukushima, Japan; ^5^Department of Innovative Research and Education for Clinicians and Trainees (DiRECT), Fukushima Medical University Hospital, Fukushima, Japan; ^6^Center for Innovative Research for Communities and Clinical Excellence (CiRC2LE), Fukushima Medical University, Fukushima, Japan; ^7^Division of Nephrology, Toho University Ohashi Medical Center, Tokyo, Japan; ^8^Division of Nephrology and Endocrinology, The University of Tokyo, Tokyo, Japan.

**Supplement Table 1. Baseline characteristics of patients with missing values**

|  | Missing  (n=1,731) | Total  (n=8,571) | MCV<90 fL  (n=1,401) | 90 ≤MCV <94 fL (n=1,535) | 94 ≤MCV <98 fL (n=2,100) | 98 ≤MCV <102 fL (n=1,828) | 102 ≤ MCV fL  (n=1,707) | p-value |
| --- | --- | --- | --- | --- | --- | --- | --- | --- |
| Age, years | 63.6 (10.0) | 62.5 (12.7) | 60.2 (12.6) | 61.2 (12.6) | 62.1 (12.7) | 63.4 (12.5) | 65.3 (12.5) | 0.79 |
| Female, % | 44.4 | 37.2 | 31.8 | 35.7 | 37.1 | 38.5 | 41.7 | 0.064 |
| BMI | 21.6 (4.3) | 20.9 (3.3) | 21.4 (3.5) | 21.2 (3.2) | 21.0 (3.2) | 20.8 (3.2) | 20.2 (3.1) | 0.24 |
| Dialysis vintage, years | 5.5 (2.2-11.4) | 5.1 (1.8-10.6) | 4.6 (1.2-10.7) | 4.5 (1.4-9.5) | 5.0 (1.7-10.3) | 5.2 (2.0-10.9) | 5.7 (2.3-11.4) | 0.014 |
| Primary renal disease, % |  |  |  |  |  |  |  | < .01 |
| Chronic glomerulonephritis | 38.9 | 45.8 | 39.8 | 39.2 | 45.5 | 49.0 | 53.2 |  |
| Nephrosclerosis | 3.7 | 5.2 | 4.2 | 4.5 | 5.7 | 5.3 | 5.9 |  |
| Diabetic nephropathy | 35.2 | 29.9 | 35.9 | 36.5 | 30.4 | 26.6 | 22.1 |  |
| Secondary glomerulonephritis | 0.0 | 1.5 | 1.7 | 1.2 | 1.3 | 1.6 | 1.6 |  |
| Interstitial nephritis | 0.0 | 2.3 | 2.5 | 2.4 | 2.1 | 2.7 | 2.1 |  |
| Others | 22.2 | 15.4 | 15.9 | 16.3 | 15.1 | 14.9 | 15.2 |  |
| Comorbidities, % |  |  |  |  |  |  |  |  |
| Diabetes mellitus | 40.7 | 33.0 | 38.8 | 39.7 | 33.4 | 29.4 | 25.7 | 0.52 |
| Cardiovascular diseases | 14.8 | 29.6 | 31.1 | 28.3 | 29.3 | 27.9 | 31.8 | < .01 |
| Cerebrovascular diseases | 13.0 | 13.8 | 14.6 | 12.8 | 13.2 | 13.5 | 14.9 | 0.30 |
| Peripheral vascular diseases | 13.0 | 14.9 | 16.9 | 15.1 | 14.0 | 13.8 | 15.2 | 0.73 |
| COPD | 1.9 | 2.6 | 3.3 | 2.2 | 2.2 | 2.7 | 2.6 | < .01 |
| Liver cirrhosis | 4.2 | 2.6 | 2.4 | 2.5 | 2.6 | 1.9 | 3.8 | 0.27 |
| Cancer | 20.4 | 9.0 | 8.1 | 8.7 | 9.2 | 9.2 | 9.4 | 0.14 |
| ESAs, % |  |  |  |  |  |  |  | 0.011 |
| rHuEPO-α (or β) | 46.0 | 66.9 | 56.5 | 63.7 | 67.5 | 69.7 | 74.0 |  |
| Darbepoetin-α | 36.0 | 18.1 | 21.8 | 20.5 | 17.6 | 16.6 | 15.5 |  |
| Epoetin-β pegol | 6.0 | 1.9 | 2.1 | 2.8 | 1.3 | 1.7 | 2.0 |  |
| Non used | 12.0 | 13.1 | 19.6 | 13.0 | 13.6 | 12.1 | 8.5 |  |
| ERI |  |  |  |  |  |  |  |  |
| rHuEPO-α (or β), IU/week/kg/g/dL | 19.9 (8.8-42.6) | 16.3 (7.2-37.3) | 17.4 (8.5-49.5) | 14.7 (7.0-35.8) | 15.3 (6.7-34.5) | 16.7 (7.3-35.5) | 17.5 (7.6-38.3) | 0.095 |
| Darbepoetin-α, μg/week/kg/g/dL | 0.036 (0.022-0.076) | 0.042 (0.026-0.071) | 0.054 (0.032-0.092) | 0.042 (0.025-0.063) | 0.039 (0.025-0.063) | 0.037 (0.023-0.062) | 0.046 (0.025-0.075) | 0.27 |
| Epoetin-β pegol, μg/week/kg/g/dL | 0.063 (0.039-0.094) | 0.046 (0.030-0.067) | 0.053 (0.039-0.067) | 0.045 (0.032-0.069) | 0.045 (0.030-0.068) | 0.043 (0.029-0.054) | 0.044 (0.029-0.072) | 0.10 |
| Intravenous iron, % | 29.2 | 27.4 | 28.8 | 26.1 | 24.1 | 28.9 | 29.9 | < .01 |
| Laboratory variables |  |  |  |  |  |  |  |  |
| MCV, fL | - | 96.1 (6.9) | 85.4 (4.0) | 91.9 (1.1) | 95.7 (1.1) | 99.5 (1.1) | 105.4 (3.1) |  |
| Hemoglobin, g/L | - | 10.2 (1.3) | 10.1 (1.5) | 10.3 (1.3) | 10.3 (1.3) | 10.3 (1.3) | 10.1 (1.3) |  |
| WBC, 10^3^/mm^3^ | - | 6.0 (1.9) | 6.4 (2.1) | 6.1 (2.0) | 5.9 (1.9) | 5.8 (1.8) | 5.6 (1.9) |  |
| TSAT, % | - | 24.4 (11.5) | 17.9 (11.6) | 22.7 (11.1) | 25.4 (10.6) | 26.9 (10.9) | 28.8 (10.3) |  |
| Ferritin, ng/mL | - | 112.7 (44.2-260) | 58 (20.4-154.5) | 90 (35.6-200) | 110 (47-238) | 140 (58-310.1) | 166 (84.5-376) |  |
| CRP, mg/dL | - | 0.12 (0.06-0.38) | 0.17 (0.08-0.47) | 0.11 (0.05-0.30) | 0.15 (0.06-0.40) | 0.10 (0.05-0.26) | 0.13 (0.06-0.46) |  |

Results of continuous variables are shown as mean (SD), or median (interquartile range). BMI, body mass index; COPD, chronic obstructive pulmonary disease; ESA, erythropoiesis stimulating agents; ERI, erythropoiesis resistance index; rHuEPO-α (or β), recombinant human erythropoietin-alpha (or beta); MCV, mean corpuscular volume; WBC, White blood cell; TSAT, Transferrin saturation; CRP, C-reactive protein.

ERI was calculated by dividing the weekly ESA dose by body weight (kg) per hemoglobin value (g per dL).

P-value testing between patients with missing values (n=1,731) and total of included patients (n=8,571) are shown.
