# Supplementary material for: Low rather than high mean corpuscular volume is associated with mortality in Japanese patients under hemodialysis
Source: Sci Rep. 2020 Sep 24;10:15663. doi: 10.1038/s41598-020-72765-2 (PMC7515877; doi:10.1038/s41598-020-72765-2)
Supplement: Supplementary file 3 — Supplementary Table 2. [file 41598_2020_72765_MOESM3_ESM.docx]

**Low rather than high mean corpuscular volume is associated with mortality in Japanese patients under hemodialysis**

Hirokazu Honda^1^, Miho Kimachi^2,3^, Noriaki Kurita^4,5,6^, Nobuhiko Joki^7^, Masaomi Nangaku^8^

^1^Department of Medicine, Division of Nephrology, Showa University School of Medicine, Tokyo, Japan; ^2^Department of Healthcare Epidemiology, School of Public Health in the Graduate School of Medicine, Koto University, Kyoto, Japan; ^3^Institute for Health Outcomes and Process Evaluation Research (iHope International), Kyoto, Japan; ^4^Department of Clinical Epidemiology, Graduate School of Medicine, Fukushima Medical University, Fukushima, Japan; ^5^Department of Innovative Research and Education for Clinicians and Trainees (DiRECT), Fukushima Medical University Hospital, Fukushima, Japan; ^6^Center for Innovative Research for Communities and Clinical Excellence (CiRC2LE), Fukushima Medical University, Fukushima, Japan; ^7^Division of Nephrology, Toho University Ohashi Medical Center, Tokyo, Japan; ^8^Division of Nephrology and Endocrinology, The University of Tokyo, Tokyo, Japan.

**Supplement Table 2．Causes of all-cause death**

| **Causes** | **Total (n=911)** | **MCV<90 fL**  **(n=180)** | **90 ≤MCV <94 fL (n=131)** | **94 ≤MCV <98 fL (n=203)** | **98 ≤MCV <102 fL (n=189)** | **102 ≤ MCV fL (n=208)** |
| --- | --- | --- | --- | --- | --- | --- |
| Cardiovascular events | 91 (10.0) | 23 (12.8) | 11 (8.4) | 20 (9.9) | 22 (11.6) | 15 (7.2) |
| Other cardiac events | 216 (23.7) | 41 (22.8) | 37 (28.2) | 47 (23.2) | 40 (21.2) | 51 (24.5) |
| Pulmonary embolism | 3 (0.33) | 0 (0) | 1 (0.76) | 0 (0) | 1 (0.53) | 1 (0.48) |
| Cerebrovascular disease | 89 (9.8) | 15 (8.3) | 17 (13.0) | 24 (11.8) | 20 (10.6) | 13 (6.3) |
| Bleeding | 7 (0.77) | 1 (0.56) | 0 (0) | 1 (0.49) | 3 (1.6) | 2 (0.96) |
| Infectious disease | 123 (13.5) | 33 (18.3) | 18 (13.7) | 22 (10.8) | 27 (14.3) | 23 (11.1) |
| Liver disease | 9 (0.99) | 2 (1.1) | 0 (0) | 1 (0.49) | 1 (0.53) | 5 (2.4) |
| Gastrointestinal disease | 25 (2.7) | 3 (1.7) | 0 (0) | 3 (1.5) | 5 (2.7) | 14 (6.7) |
| Chronic obstructive pulmonary disease | 1 (0.11) | 0 (0) | 0 (0) | 0 (0) | 1 (0.53) | 0 (0) |
| Cancers | 76 (8.3) | 8 (4.4) | 14 (10.7) | 20 (9.9) | 16 (8.5) | 18 (8.7) |
| Others | 87 (9.6) | 16 (8.9) | 11 (8.4) | 22 (10.8) | 20 (10.6) | 18 (8.7) |
| Unknown | 184 (20.2) | 38 (21.1) | 22 (16.8) | 43 (21.2) | 33 (17.5) | 48 (23.1) |

MCV, mean corpuscular volume. Results are shown as number of deaths with percentage in each category.
