# Supplementary material for: Low rather than high mean corpuscular volume is associated with mortality in Japanese patients under hemodialysis
Source: Sci Rep. 2020 Sep 24;10:15663. doi: 10.1038/s41598-020-72765-2 (PMC7515877; doi:10.1038/s41598-020-72765-2)
Supplement: Supplementary file 5 — Supplementary Table 6. [file 41598_2020_72765_MOESM5_ESM.docx]

**Low rather than high mean corpuscular volume is associated with mortality in Japanese patients under hemodialysis**

Hirokazu Honda^1^, Miho Kimachi^2,3^, Noriaki Kurita^4,5,6^, Nobuhiko Joki^7^, Masaomi Nangaku^8^

^1^Department of Medicine, Division of Nephrology, Showa University School of Medicine, Tokyo, Japan; ^2^Department of Healthcare Epidemiology, School of Public Health in the Graduate School of Medicine, Koto University, Kyoto, Japan; ^3^Institute for Health Outcomes and Process Evaluation Research (iHope International), Kyoto, Japan; ^4^Department of Clinical Epidemiology, Graduate School of Medicine, Fukushima Medical University, Fukushima, Japan; ^5^Department of Innovative Research and Education for Clinicians and Trainees (DiRECT), Fukushima Medical University Hospital, Fukushima, Japan; ^6^Center for Innovative Research for Communities and Clinical Excellence (CiRC2LE), Fukushima Medical University, Fukushima, Japan; ^7^Division of Nephrology, Toho University Ohashi Medical Center, Tokyo, Japan; ^8^Division of Nephrology and Endocrinology, The University of Tokyo, Tokyo, Japan.

**Supplement Table 6. Association of MCV with iron supplementation, biomarker for iron metabolism and C-reactive protein**

|  | **MCV, fL**  **β coefficient (95% CI)** | **p-value** |
| --- | --- | --- |
| Intravenous iron, % | 0.052 (-0.011 to 0.11) | 0.11 |
| Transferrin saturation, % | 0.0037 (0.0015 to 0.0059) | 0.002 |
| Ferritin, ng/mL | 0.00017 (0.00012 to 0.00021) | < 0.001 |
| C-reactive protein, mg/dL | -0.00061 (-0.0020 to 0.00078) | 0.37 |

MCV, mean corpuscular volume

We have assessed the association between continuous MCV level and iron supplementation, transferrin saturation, ferritin and C-reactive protein using a linear regression model with robust standard variance estimation to account for facility clustering.
