# Supplementary material for: Low rather than high mean corpuscular volume is associated with mortality in Japanese patients under hemodialysis
Source: Sci Rep. 2020 Sep 24;10:15663. doi: 10.1038/s41598-020-72765-2 (PMC7515877; doi:10.1038/s41598-020-72765-2)
Supplement: Supplementary file 6 — Supplementary Table 7–10. [file 41598_2020_72765_MOESM6_ESM.docx]

**Low rather than high mean corpuscular volume is associated with mortality in Japanese patients under hemodialysis**

Hirokazu Honda^1^, Miho Kimachi^2,3^, Noriaki Kurita^4,5,6^, Nobuhiko Joki^7^, Masaomi Nangaku^8^

^1^Department of Medicine, Division of Nephrology, Showa University School of Medicine, Tokyo, Japan; ^2^Department of Healthcare Epidemiology, School of Public Health in the Graduate School of Medicine, Koto University, Kyoto, Japan; ^3^Institute for Health Outcomes and Process Evaluation Research (iHope International), Kyoto, Japan; ^4^Department of Clinical Epidemiology, Graduate School of Medicine, Fukushima Medical University, Fukushima, Japan; ^5^Department of Innovative Research and Education for Clinicians and Trainees (DiRECT), Fukushima Medical University Hospital, Fukushima, Japan; ^6^Center for Innovative Research for Communities and Clinical Excellence (CiRC2LE), Fukushima Medical University, Fukushima, Japan; ^7^Division of Nephrology, Toho University Ohashi Medical Center, Tokyo, Japan; ^8^Division of Nephrology and Endocrinology, The University of Tokyo, Tokyo, Japan.

**Supplement Table 7. Association between MCV and death due to cardiovascular events**

|  | **Model 1 HR** | **p-value** | **Model 2 HR** | **p-value** | **Model 3 HR** | **p-value** |
| --- | --- | --- | --- | --- | --- | --- |
| Low (MCV < 90 fL) | 1.84 (0.96 to 3.54) | 0.067 | 1.84 (0.95 to 3.53) | 0.068 | 1.75 (0.88 to 3.47) | 0.11 |
| Slightly low (90 ≤ MCV < 94 fL) | 0.78 (0.39 to 1.53) | 0.45 | 0.76 (0.38 to 1.52) | 0.44 | 0.75 (0.38 to 1.51) | 0.42 |
| Medium (94 ≤ MCV < 98 fL) | Reference |  | Reference |  | Reference |  |
| Slightly high (98 ≤ MCV < 102 fL) | 1.24 (0.65 to 2.37) | 0.52 | 1.24 (0.65 to 2.37) | 0.52 | 1.24 (0.64 to 2.39) | 0.52 |
| High (102 ≤ MCV fL) | 0.92 (0.45 to 1.89) | 0.81 | 0.92 (0.45 to 1.89) | 0.81 | 0.88 (0.44 to 1.75) | 0.71 |

HR, hazard ratio; MCV, mean corpuscular volume. Results are shown as HR with 95% confidence intervals.

Adjusted for

- Model 1 (minimally adjusted model): levels of ferritin, transferrin saturation (TSAT)
- Model 2: levels of ferritin, TSAT and C-reactive protein (CRP)
- Model 3 (fully adjusted model): all potential risk factors at baseline, including age, gender, body mass index, dialysis vintage, primary renal disease, comorbidities (diabetes mellitus, cardiovascular diseases, cerebrovascular diseases, peripheral atherosclerotic vascular diseases, chronic obstructive pulmonary disease, liver cirrhosis, and cancer), laboratory values (hemoglobin, white blood cells, TSAT, ferritin and CRP), administration of erythropoiesis-stimulating agents and intravenous iron infusion.

**Supplement Table 8. Association between MCV and death due to infection**

|  | **Model 1 HR** | **p-value** | **Model 2 HR** | **p-value** | **Model 3 HR** | **p-value** |
| --- | --- | --- | --- | --- | --- | --- |
| Low (MCV < 90 fL) | 2.36 (1.35 to 4.12) | 0.003 | 2.35 (1.34 to 4.11) | 0.003 | 2.21 (1.22 to 3.99) | 0.009 |
| Slightly low (90 ≤ MCV < 94 fL) | 1.14 (0.59 to 2.21) | 0.71 | 1.13 (0.58 to 2.20) | 0.72 | 1.13 (0.59 to 2.19) | 0.71 |
| Medium (94 ≤ MCV < 98 fL) | Reference |  | Reference |  | Reference |  |
| Slightly high (98 ≤ MCV < 102 fL) | 1.38 (0.80 to 2.37) | 0.24 | 1.38 (0.80 to 2.36) | 0.25 | 1.15 (0.67 to 1.98) | 0.62 |
| High (102 ≤ MCV fL) | 1.27 (0.68 to 2.35) | 0.46 | 1.27 (0.68 to 2.35) | 0.46 | 0.89 (0.47 to 1.70) | 0.74 |

HR, hazard ratio; MCV, mean corpuscular volume. Results are shown as HR with 95% confidence intervals.

Adjusted for

- Model 1 (minimally adjusted model): levels of ferritin, transferrin saturation (TSAT)
- Model 2: levels of ferritin, TSAT and C-reactive protein (CRP)
- Model 3 (fully adjusted model): all potential risk factors at baseline, including age, gender, body mass index, dialysis vintage, primary renal disease, comorbidities (diabetes mellitus, cardiovascular diseases, cerebrovascular diseases, peripheral atherosclerotic vascular diseases, chronic obstructive pulmonary disease, liver cirrhosis, and cancer), laboratory values (hemoglobin, white blood cells, TSAT, ferritin and CRP), administration of erythropoiesis-stimulating agents and intravenous iron infusion.

**Supplement Table 9. Association between MCV and death due to cardiovascular events using time-varying Cox regression analysis**

|  | **Model 1 HR** | **p-value** | **Model 2 HR** | **p-value** | **Model 3 HR** | **p-value** |
| --- | --- | --- | --- | --- | --- | --- |
| Low (MCV < 90 fL) | 1.39 (0.77 to 2.51) | 0.27 | 1.39 (0.77 to 2.51) | 0.27 | 1.36 (0.76 to 2.41) | 0.30 |
| Slightly low (90 ≤ MCV < 94 fL) | 0.88 (0.42 to 1.86) | 0.75 | 0.89 (0.42 to 1.87) | 0.75 | 0.90 (0.43 to 1.88) | 0.78 |
| Medium (94 ≤ MCV < 98 fL) | Reference |  | Reference |  | Reference |  |
| Slightly high (98 ≤ MCV < 102 fL) | 1.19 (0.62 to 2.30) | 0.60 | 1.20 (0.62 to 2.31) | 0.60 | 1.21 (0.61 to 2.38) | 0.59 |
| High (102 ≤ MCV fL) | 0.85 (0.44 to 1.62) | 0.62 | 0.85 (0.44 to 1.62) | 0.62 | 0.90 (0.47 to 1.74) | 0.76 |

HR, hazard ratio; MCV, mean corpuscular volume. Results are shown as HR with 95% confidence intervals. We performed time-varying Cox regression analysis to account for fluctuation in MCV during the follow-up period.

Adjusted for

- Model 1 (minimally adjusted model): time-varying covariates every 4 months, including levels of ferritin, transferrin saturation (TSAT)
- Model 2: time-varying covariates every 4 months, including levels of ferritin, TSAT and C-reactive protein (CRP)
- Model 3 (fully adjusted model): baseline characteristics, including patient demographics (age, gender, body mass index, dialysis vintage, primary renal disease), comorbidities (diabetes mellitus, cardiovascular disease, cerebrovascular disease, peripheral atherosclerotic vascular disease, chronic obstructive pulmonary disease, liver cirrhosis, and cancer), and for time-varying covariates every 4 months, including laboratory measurements (hemoglobin, white blood cells, TSAT, ferritin and CRP) and administration of erythropoiesis-stimulating agents and intravenous iron infusion.

**Supplement Table 10. Association between MCV and death due to infection using time-varying Cox regression analysis**

|  | **Model 1 HR** | **p-value** | **Model 2 HR** | **p-value** | **Model 3 HR** | **p-value** |
| --- | --- | --- | --- | --- | --- | --- |
| Low (MCV < 90 fL) | 3.17 (1.40 to 7.21) | 0.006 | 3.19 (1.40 to 7.26) | 0.006 | 3.41 (1.52 to 7.65) | 0.003 |
| Slightly low (90 ≤ MCV < 94 fL) | 2.55 (1.27 to 5.13) | 0.008 | 2.56 (1.27 to 5.15) | 0.008 | 2.78 (1.39 to 5.58) | 0.004 |
| Medium (94 ≤ MCV < 98 fL) | Reference |  | Reference |  | Reference |  |
| Slightly high (98 ≤ MCV < 102 fL) | 0.84 (0.35 to 2.01) | 0.70 | 0.84 (0.35 to 2.02) | 0.70 | 0.73 (0.30 to 1.74) | 0.47 |
| High (102 ≤ MCV fL) | 2.71 (1.39 to 5.28) | 0.003 | 2.72 (1.39 to 5.31) | 0.003 | 2.25 (1.12 to 4.53) | 0.024 |

HR, hazard ratio; MCV, mean corpuscular volume. Results are shown as HR with 95% confidence intervals. We performed time-varying Cox regression analysis to account for fluctuation in MCV during the follow-up period.

Adjusted for

- Model 1 (minimally adjusted model): time-varying covariates every 4 months, including levels of ferritin, transferrin saturation (TSAT)
- Model 2: time-varying covariates every 4 months, including levels of ferritin, TSAT and C-reactive protein (CRP)
- Model 3 (fully adjusted model): baseline characteristics, including patient demographics (age, gender, body mass index, dialysis vintage, primary renal disease), comorbidities (diabetes mellitus, cardiovascular disease, cerebrovascular disease, peripheral atherosclerotic vascular disease, chronic obstructive pulmonary disease, liver cirrhosis, and cancer), and for time-varying covariates every 4 months, including laboratory measurements (hemoglobin, white blood cells, TSAT, ferritin and CRP) and administration of erythropoiesis-stimulating agents and intravenous iron infusion.
